# A Reinforcement Learning Model for Optimal Treatment Strategies in Intensive Care: Assessment of the Role of Cardiorespiratory Features

**DOI:** 10.1109/OJEMB.2024.3367236

**Published:** 2024-02-19

**Authors:** Cristian Drudi, Maximiliano Mollura, Li-wei H. Lehman, Riccardo Barbieri

**Affiliations:** Department of Electronics, Informatics and EngineeringPolitecnico di Milano 18981 20133 Milano Italy; Institute for Medical Engineering and ScienceMassachusetts Institute of Technology 2167 Cambridge MA 02139 USA

**Keywords:** Cardiovascular system, dimensionality reduction, intensive care unit, reinforcement learning, sepsis

## Abstract

*Goal:* The purpose of this study is to evaluate the importance of cardiorespiratory variables within a Reinforcement Learning (RL) recommendation system aimed at establishing optimal strategies for drug treatment of septic patients in the intensive care unit (ICU). *Methods:* We developed a RL model in order to establish drug administration strategies for septic patients using only a set of cardiorespiratory variables. We then compared this model with other RL models trained with a different set of features. We selected patients meeting the Sepsis-3 criteria from the Multi-parameter Intelligent Monitoring in Intensive Care (MIMIC III) database, resulting in a total of 20,496 ICU admissions. A Markov Decision Process (MDP) was built on the extracted discrete time-series. A policy iteration algorithm was used to obtain the optimal AI policy for the MDP. The policy performance was then evaluated using the WIS estimator. The process was repeated for each set of variables and compared to a set of baseline benchmark policies. *Results:* The model trained with cardiorespiratory variables outperformed all other models considered, resulting in a 95% confidence lower bound score of 97.48. This finding highlights the importance of cardiovascular variables in the clinical RL recommendation system. *Conclusions:* We established an efficient RL model for sepsis treatment in the ICU and demonstrated that cardiorespiratory variables provides critical information in devising optimal policies. Given the potentially continuous availability of cardiorespiratory features extracted from bedside physiological waveform monitoring, the proposed framework paves the way for a real time recommendation system for sepsis treatment.

## I. Introduction

In The last decades, research in healthcare is focusing its interest on the application of advanced statistical modeling techniques to develop decision support systems [1], [2]. This phenomenon has been made possible thanks to the increasing ability to collect, store and process a large quantity of clinical data from real-world scenarios [3], [4]. Usually, in critical care practice, large amounts of granular data are generated and stored in electronic health records. In this context, several studies have already proposed the application of artificial intelligence (AI) tools, showing their ability to timely identify a wide range of medical conditions. Specific successful applications are devoted to the prediction of complications, mortality prediction, improvement of prognostic models, and classification of sub-populations [5], together with the identification of sepsis in the early hours of intensive care unit (ICU) admission [6], the detection of congestive heart failure (CHF) [7], and the identification of coronavirus disease 2019 (COVID-19) [8]. AI models also proved their ability in predicting the evolution of diseases over the course of the ICU stay. Chen et al. [9] developed a model able to predict events of cardiorespiratory health deterioration 90 minutes prior to the crisis, and Nemati et al. [10] managed to develop a model able to predict the onset of sepsis 4 to 12 hours prior to the clinical recognition. AI also demonstrated the ability to discern different phenotypes of sepsis as shown by Seymour et al. [11] and Calfee et al. [12] were able to identify two different subtypes of respiratory distress syndrome (ARDS), linked to different clinical outcomes and specific treatment responses.

Recently, several solutions have proposed the application of a sub-area of Machine Learning (ML) called Reinforcement Learning (RL), particularly focused on the development of decision-making agents, which could further exploit such a huge amount of generated data [13]. Some examples in this field are: the tracking of optimal glycemic level [14], the optimization of weaning time from mechanical ventilation [15], and the continuous optimization of morphine dosage [16].

The popularity of the RL framework derives from the possibility to estimate a treatment policy that maximizes or minimizes the probability of a specific clinical outcome (e.g. mortality) and, at the same time, it allows for the assessment of current treatment strategies by exploring the different treatment options that are commonly made by clinicians. Indeed, these novel tools would also be used to evaluate the potential of variables directly related to real physiological mechanisms to provide useful information to the model, thus enriching our clinical knowledge.

Several studies have recently applied RL to improve the treatment of patients with sepsis in the ICU [17], [18], [19]. In particular, Komorowski et al. [18] developed an RL agent able to optimally dosage vasopressors and intravenous fluids for septic ICU patients, addressing the lack of tailored treatments accounting for the individuality of patients.

Sepsis is one of the major causes of death in the world, causing 8 million deaths every year. The mortality of sepsis is usually between 15% and 30%, spiking to 50% and higher in the case of developing countries [20]. Sepsis has a high impact also by a financial standpoint. In 2013 the most expensive condition in the USA was septicemia carrying 6.2% of United States national costs, totaling an amount of $23.7 billion [21]. Despite the tangible impact of sepsis, common consensus in treating sepsis is still absent. There is controversy around many variables regarding the management of sepsis, and agreement is shared by clinicians just around a few key points [22]. Prompt initiation of treatment with vasopressors and intravenous fluids is of central importance to address hypotension and tissue hypoperfusion induced by septic shock. These treatments are very important, especially in the first hour after sepsis identification, as indicated in the Surviving Sepsis Campaign (SSC) Guidelines [23]. However, clinical guidelines only offer a broad treatment approach for ICU patients with sepsis. Personalized strategies based on the patient's specific attributes and responses to treatment are yet to be established. Recent research into developing an AI policy through reinforcement learning has shown promising results in this context [18]. According to the authors, their policy was successful in reducing the 90-day mortality rate in the intensive care unit for patients with sepsis by administering individualized doses of vasopressors and intravenous fluids customized for each patient's condition.

Nowadays, many of the studies employing RL in healthcare contexts rely on the definition of a Markov Decision Process (MDP) to model the clinical evolution of the patient during his stay in the ICU. MDPs usually require distance-based methods (e.g., clustering algorithms) to summarize the information describing the patient state. However, due to the large set of features employed to fully describe the patient state in the ICU environment, clustering algorithms would suffer from such a high dimensional feature space because of the curse of dimensionality [24]. Moreover, a high dimensionality also increases the computational cost required by the clustering algorithm.

Our study reproduces and further analyzes the solution proposed by Komorowski et al. [18] by focusing on different dimensionality reduction approaches. In particular, we explore the performance of the resulting AI agent when using a subset of cardiovascular variables.

This is of particular interest because the cardiovascular variables are always steadily available in the critical care ward since they are constantly collected at the bedside by specific monitoring equipment, while other variables employed in previous studies [17], [18] like the laboratory values are sparse in time and their results require hours to be available. In a real-world setting, this would hinder the development of a real-time recommendation system since laboratory values would never be available immediately.

Cardiovascular variables have also a potentially high information content since a wide range of additional features can be extracted from the physiological signals related to the cardiovascular system and these features can be used by the model to further increase its performance.

## II. Materials and Methods

### A. Data Source and Patient Cohort

This study is conducted on the “Multi-parameter Intelligent Monitoring in Intensive Care” (MIMIC III) database [25]. MIMIC-III is a collection of data from the Beth Israel Deaconess Medical center (BIDMC) in Boston, MA. This database contains data from 53,423 distinct hospital admissions for patients aged 16 years or above, collected between 2001 and 2012.

The cohort is composed of patients that fulfill the sepsis-3 criteria [26]. The following exclusion criteria are also applied: exclusion of patients that at the time of admission were younger than 18 years old; exclusion of patients whose mortality or intravenous fluid administration were not documented; exclusion of patients whose treatment was withdrawn, thus excluding unrecoverable patients or under palliative care. In this work, patient withdrawal is defined as a patient who died within 24 hours from the end of data recording and did not receive any vasopressor for the last 24 hours recorded, but received at least one administration previously. The final number of unique ICU stays included in this study is 20,496.

Sepsis was identified according to two possible criteria, which include either the presence of a microbiological sampling before an antibiotic administration or a microbiological sampling within 24 hours from a previous antibiotic administration. In the first case, sepsis onset is defined only if the antibiotic is given no later than 72 hours from the microbiological sampling. The sepsis onset timing is defined as the time of the earliest event [27].

The data included in this study temporally range from 24 hours before the estimated onset of sepsis to 48 hours from the estimated onset of sepsis. The data are organized as time series with 4-hour time bins. If multiple observations for the same variable were available at a given time bin, they were averaged or summed accordingly to the type of variable. Forty-eight variables are selected, some examples are: vital signs, demographics, laboratory values, fluid balance, the Elixhauser comorbidity score [28], and the treatments of interest: intravenous fluids and vasopressors. A baseline zero SOFA score assumption was made in line with previous work in the literature [29]. Variables with heterogeneous units of measures were uniformed and the numerical values of the variable are capped to plausible physiological values.

A zero-order interpolation approach [30] is used to address the sparsity of data, the use of this technique is frequent when dealing with clinical time series and also matches the behavior of medical decision-making [31]. The remaining missing data is imputed through interpolation. 80% of the available data is used for training and 20% for testing.

**Fig. 1.**
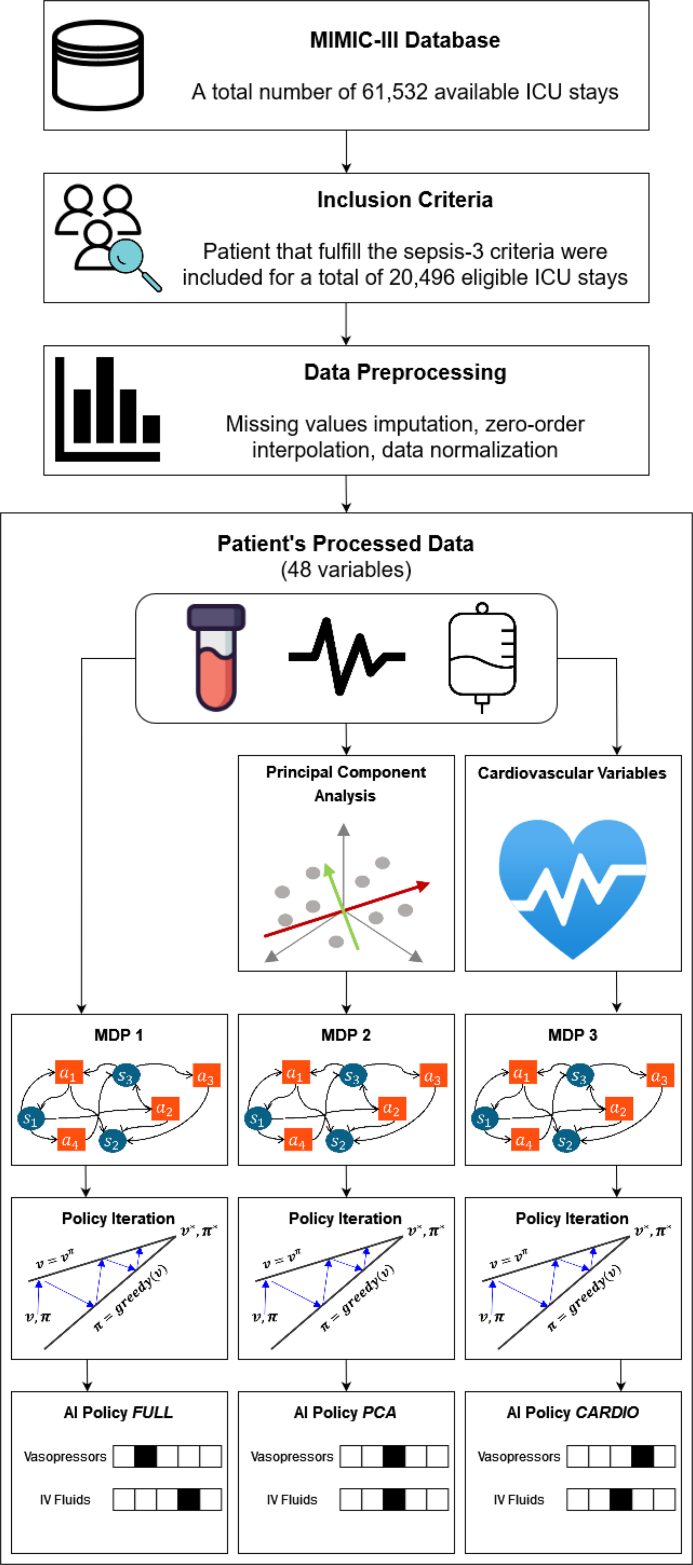
Flowchart of the steps performed in this study.

Fig. 1 illustrates the workflow of this work.

### B. Developed Reinforcement Learning Models

In order to investigate the role of different approaches for reducing the feature space, we first assessed the performance of the method proposed by Komorowski et al., referred to as *FULL* Model, which includes 48 features. Successively, we compared the obtained results with the performance of a series of different models trained on specific subsets of features, or trained by limiting the action space of the agent. The following additional models were proposed:

*Dimensionality reduction techniques:* The feature space is reduced by applying principal component analysis (PCA) on the full set of features. Fig. 5 shows the loadings of the first principal component. Two distinct subsets of features were considered.

**Fig. 2.**
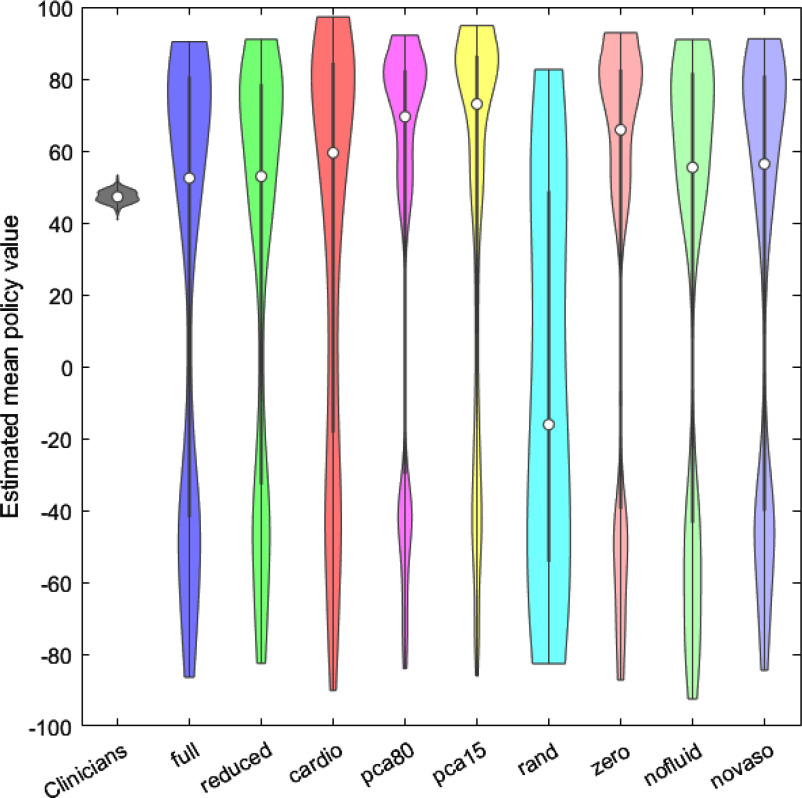
Mean policy values violin plots.

**Fig. 3.**
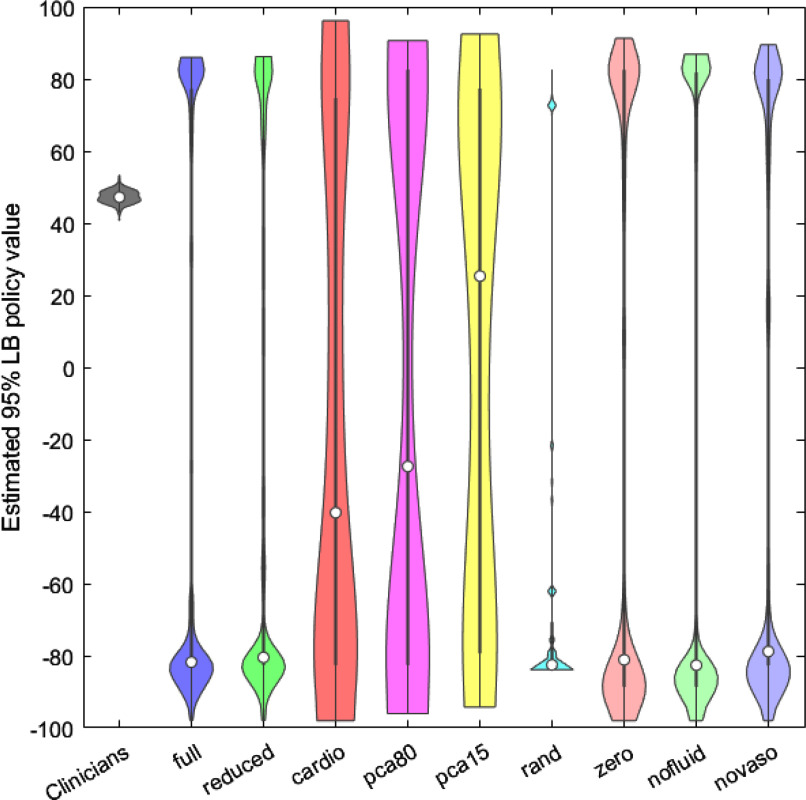
95% policy values lower bound violin plots.

**Fig. 4.**
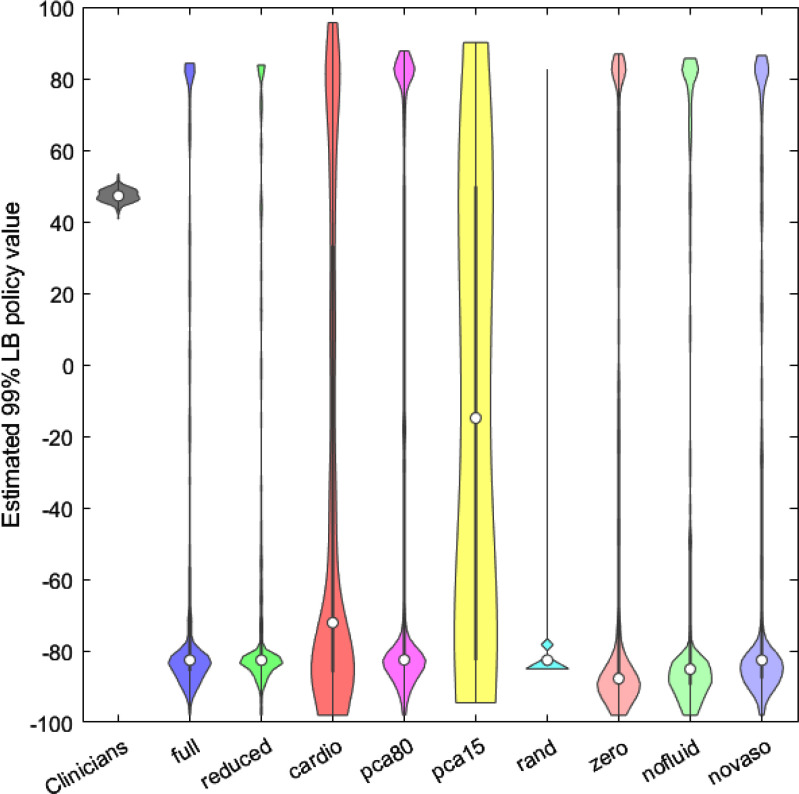
99% policy values lower bound violin plots.

**Fig. 5.**
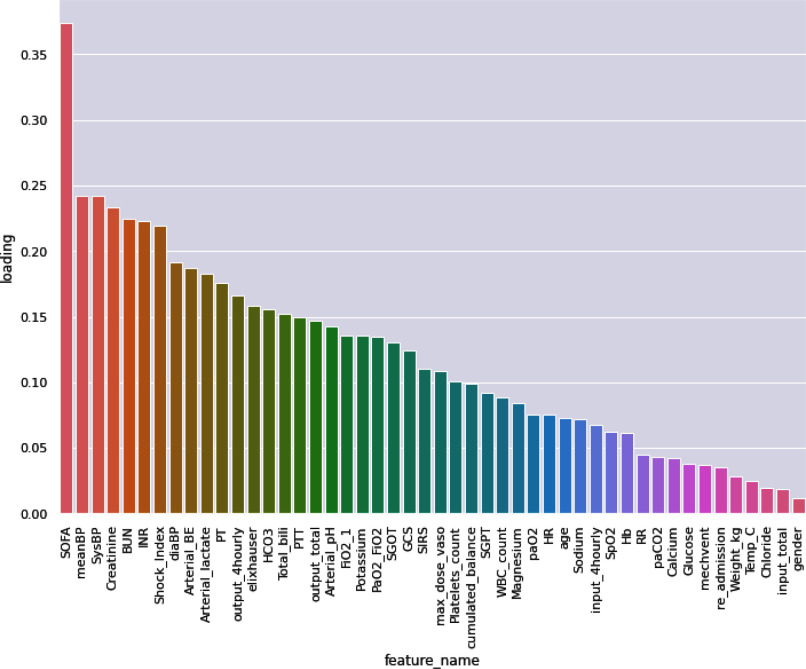
First principal component loadings.

• *PCA 80%:* This subset includes only the most important features explaining 80% of the variance, thus resulting in the first 25 principal components.
• *PCA 15%:* This subset is reduced to the first 2 principal components only, which explain about 15% of the variance.

*A-priori feature selection:* The second approach consists of an a-priori reduction of the feature space based on some practical considerations.

• *NO LAB:* This subset excludes features extracted from the laboratory analysis, which usually incurs in a delay between their sampling and the availability of the results. In this case, the feature subset includes 22 features.
• *CARDIO:* The second subset of the a-priori approach includes a set of variables closely linked to the cardiovascular system functioning, as it is supposed to be strongly influenced by the treatments under analysis. In particular, heart rate, systolic blood pressure, diastolic blood pressure, shock index, $SpO_{2}$, and mechanical ventilation are considered
• *BASELINE:* The last subset is mainly used as a reference and includes only the 6 features with the lowest importance according to the PCA, thus providing a comparative measure for the reduced models. Considered features are: gender, total fluid input, chloride, body temperature, weight, and readmission.

*Policy constrained models:* We compared the performance of the developed policies with a set of policy-constrained models.

• *RAND:* Policy that takes action randomly.
• *NO DRUGS:* Policy that never provide any treatment.
• *NO VASOPRESSORS:* Policy that never provide vasopressors.
• *NO FLUID:* Policy that never provide intravenous fluids.

Finally, since a reduced feature space might imply that a reduced number of clusters is more optimal, we retrained the models that underwent feature space dimensionality reduction with a number of clusters equal to 250.

### C. Markov Decision Process

Mathematical models for solving sequential decision problems generally employ Markov decision processes (MDPs). An MDP comprises a tuple $M = (S, A, T, d_{0}, r, \gamma)$, in which:

• *S* denotes a set of states *s* $\in$ *S*, that might be either discrete or continuous;
• *A* is a set of actions *a* $\in$ *A*, that can also be either discrete or continuous;
• $T$ defines the system's dynamics by outlining a conditional probability distribution in the form of $T(s^{\prime },s,a)$;
• $d_{0}$ specifies the initial state distribution $d_{0}(s_{0})$;
• $r: S \times A \rightarrow \mathbb {R}$ represents the function that defines the rewards;
• $\gamma$ is a scalar discount factor.

In order to build a Markov Decision Process on the considered dataset, each patient is assigned to a state by assigning its clinical variables to a cluster determined on the train set with a k-means++ algorithm, where each cluster represents a distinct state. We chose a value of k equal to 750. This results in a total of 750 states, plus two absorbing states that represent either the survival or death of the patient. This choice was made to ensure comparability with the study conducted by Komorowski et al. [18].

The actions are defined by grouping the available treatments in IV fluid and vasopressor data, resulting in a total of five bins per treatment type. One bin is designated exclusively for null dosage, while the edges of the remaining four bins are determined by the 25th, 50th (median), and 75th percentiles of the treatment distribution. Therefore, considering all possible combinations between the two types of treatments, the total number of feasible actions amounts to 25. Our model's reward mechanism is solely based on 90-day mortality. When the Markov Decision Process (MDP) terminates in the ‘survived' state, the agent is allocated a positive reward of +100. Conversely, if the MDP concludes in the ’dead' state, the agent receives a negative reward (a ’penalty' of −100). This design choice is intentional. It aims to encourage the reinforcement learning (RL) agent to prioritize and maximize only 90-day survival. This design allows the model to avoid making assumptions about the optimal physiological values to maintain, as these values may vary between patients. The dynamic evolution of the MDP is determined by the transition matrix $T(s^{\prime },s,a)$, which defines the probability of each state-action-new state (*s-a-s'*) triplets. Transitions that occurred less than 5 times were excluded, thus forcing the RL agent to select only actions commonly selected by clinicians, thus making the resulting policy safer.

### D. Optimal Policy and Evaluation

The policy iteration algorithm was used to solve the MDP. The algorithm starts with an initial arbitrary policy that is evaluated by computing the policy value $V^{\pi }(s)$ and then the policy is improved by updating the optimal action for each state. This process has to be repeated until we converge to the optimal policy [32].

$Q^\pi (s,a)$ (state-action value function) represents the expected sum of discounted rewards of a policy $\pi$, given a state-action pair (*s,a*) and it represents how good is taking action *a* starting from state *s*.

The evaluation of the clinician's policy was performed with temporal difference (TD) learning, using the following formulation:

\begin{equation*}
Q^\pi (s,a) \leftarrow Q^\pi (s,a) + \alpha (r+\gamma Q^\pi (s^{\prime },a^{\prime })-Q^\pi (s,a)) \tag{1}
\end{equation*}

where $\alpha$ indicates the learning rate, $r$ is the immediate reward, *s'* is the future state, and *a'* the future action.

The optimal policy determined by policy iteration was defined as:

\begin{equation*}
\pi (s)^* \leftarrow \text{argmax}_{a} Q^{\pi ^*}(s,a) \forall s \tag{2}
\end{equation*}

Since in our models the reward is solely linked to mortality, the model theoretically minimize mortality rates.

Therefore, when following policy $\pi$, the state-value of each state $s$ can be determined using the Bellman equation:

\begin{equation*}
V^{\pi }(s) = \sum _{a} \pi (s,a) \sum _{s^{\prime }} T(s^{\prime },s,a) [R(s^{\prime })+\gamma V^{\pi }(s^{\prime }) ] \tag{3}
\end{equation*}

We performed off-policy evaluation (OPE) to assess the value of AI policies using patient trajectories generated by clinicians' policies. For this purpose, we employed a widely used OPE method called weighted importance sampling (WIS) [32]. Although WIS is a biased estimator, it has the desirable property of consistency and literature suggests that this characteristic is preferable to unbiasedness [33], [34]. Importance sampling methods are often used to correct for differences between the data-generating behavioral policy and the policy being evaluated. In addition, the use of WIS helps to reduce the variance of the estimates compared to unweighted importance sampling approaches. Bootstrapping was used to assess the confidence intervals of the policy value.

The cumulative importance ratio computed up to time step $t$ is:

\begin{equation*}
\rho _{1:t} := \prod _{t^{\prime }=1}^{t} \pi _{AI}(a_{t^{\prime }}\Vert s_{t^{\prime }})/\pi _{C}(a_{t^{\prime }}\Vert s_{t^{\prime }}) \tag{4}
\end{equation*}

where $\pi _{C}$ represents the clinicians' policy and $\pi _{AI}$ represents the AI policy.

Its average at time $t$ is $w_{t}=\sum _{i=1}^{N} \rho _{1:t}^{}(i)/N$, where N indicates the total number of trajectories.

The WIS estimator of the single trajectory is $V_{WIS}=\frac{\rho _{1:T}}{w_{T}}\sum _{t=1}^{T} \gamma ^{t-1}r_{t}$, after computing $V_{WIS}$ for every trajectory the average of all $V_{WIS}$ determines the overall WIS estimator:

\begin{equation*}
WIS=\frac{1}{N}\sum _{i=1}^{N} V_{WIS}^{(i)} \tag{5}
\end{equation*}

## III. Results

In order to assess the effectiveness of the AI policies developed to optimize treatment dosages, we visualized the distribution of the obtained AI policy values with violin plots.

Fig. 2 shows the violin plots of the 250 mean policy values of all the developed models and the clinicians' policy.

The distribution of mean policy values obtained by the clinicians' policy over the 250 trials is confined to a narrower range and an upper value of 53.3407.

The distributions of the mean policy values obtained by the AI policies range over a wider span of values. The best AI policy obtained by the *FULL* model scored a mean policy value of 90.3975.

The *NO LAB* model with fewer features scored a top mean policy value of 91.0551, the *CARDIO* model with just 6 features obtained the highest top mean policy values out of all models with a value of 97.2608. Also, models developed on the principal components analysis obtained a better top mean policy value than the *FULL* model, the *PCA 80%* model scored 92.1578 and the *PCA 15%* model scored 94.8487. The *RAND* model obtained a top value of 82.6197 and a uniform distribution of the 250 mean policy values. The *NO DRUGS* model achieved a top mean policy value of 92.8755, the *NO FLUID* model obtained 90.9727 and the *NO VASOPRESSORS* got a value of 91.1737.

Fig. 3 shows the violin plots of the 250 95% policy values lower bounds (LB) of all the developed models and the clinicians' policy.

The clinicians policy 95% LB has a behaviour similar to the mean policy value, it is confined to a narrow range of values and the best trial achieves a score of 53.3407.

The median of the distribution of the AI policy values shifted to negative values the exception was the *PCA 15%* model. The *CARDIO* and the *PCA 80%* models however have a median of the 95% LB higher than average and a better distribution of the values than the other models.

The best-performing model was the *CARDIO* model, with a top 95% LB policy value of 96.1722. *PCA 15%* and *PCA 80%* achieved values of 92.4912 and 90.6565. *FULL* and *NO LAB* models achieved a top 95% LB policy value of 86.0051 and 86.2196. The *RAND* model presented the worst distribution of the 95% LB and a top value of 82.6169. *NO DRUGS*, *NO FLUID* and *NO VASOPRESSORS* achieved 91.3047, 86.8949 and 89.5337.

Fig. 4 shows violin plots of the 250 99% policy values LB of all the developed models and the clinicians' policy.

The *CARDIO* model was found to be the best performing model also in this case, with a 99% lower bound of 95.6502. This results clearly suggests a marked importance of the cardiovascular variables.

To further validate the importance of cardiovascular variables, we analyzed the loadings of the first principal component. Systolic Blood Pressure has the third highest loading, with a value of 0.241907, the Shock Index shows a loading of 0.219587, with the Diastolic Blood Pressure index coming right after with a loading of 0.191688.

Mean Heart Rate ranked lower on the list, with a loading of 0.075276, although the information regarding Heart Rate is also contained in the Shock Index.

To further validate the importance of the cardiovascular variables we trained a *BASELINE* model on the six features with the lower loadings on the first principal component. This was performed to validate the fact that the increase in performance is not just due the reduced dimensionality but because the features chosen for the *CARDIO* model are more significant and enhance the performance of the AI policy.

Table I shows the best values obtained by the *CARDIO* model and by the *BASELINE* model. The *CARDIO* model was the best performer by all the points of view with a higher mean policy value and a higher policy lower bounds indicating also a safer resulting policy.

**TABLE I:** Comparison of *CARDIO* Model Against *BASELINE* Model

Metric	*CARDIO*	*BASELINE*
Mean Policy Value	97.2608	90.9569
95% LB	96.1722	87.3243
99% LB	95.6502	84.69

To verify the robustness of the feature reduction process we retrained the models that entailed a reduction of feature dimensionality by reducing the number of clusters to 250. This is done because a reduced feature dimensionality needs a reduced set of clusters in order to obtain a better clustering solution. After retraining the models, the RL policies for all the new models were evaluated and compared to their equivalent with 750 clusters.

The *PCA 15%* model obtained with 250 clusters outperforms its equivalent obtained with 750 clusters, obtaining a 95% confidence LB of 95.0335, while the *PCA 80%* model with 250 clusters obtains a 95% LB of 93.1078 outperforming its counterpart.

The *NO LAB* policy trained with 250 clusters achieved a 95% policy value confidence LB of 93.1078, thus performing better than its 750 clusters counterpart.

The *CARDIO* model trained with 250 clusters achieved a 95% policy value confidence LB of 97.4811, obtaining the best performance out of all the models presented in this study.

A summary of the performances of the models analyzed is shown in Table II.

**TABLE II:** Comparison of the 95% Confidence LB of Models Trained With 750 Clusters and 250 Clusters

Model	750 clusters	250 clusters
*CARDIO*	96.1722	97.4811
*NO LAB*	86.2196	87.7517
*PCA 80%*	90.6565	93.1078
*PCA 15%*	92.4912	95.0335

Figures illustrating in detail the evolution of the models summarized in Table II can be found in the supplementary materials.

An higher policy value is associated to a lower mortality rate that can be retrieved through a calibration curve. A score of 80 is associated with a mortality risk of 15%, while a score of 90 is associated with a mortality risk of 8.55% and a score of 96 with a mortality risk of 3.62%.

## IV. Discussion

Many authors have used clustering approaches to assign a state to each patient observation [14], [18]. In the case of RL techniques applied to ICU data, this unsupervised approach is particularly helpful due to the complexity of properly defining a patient's state representation in the clinical context, commonly influenced by several possible confounders. However, the high dimensionality of the feature space represents one of the major limitations of partitioning clustering approaches. A high number of features has two major drawbacks: first, it significantly increases the overall computational cost of the model; and second, the curse of dimensionality kicks in, thus affecting the quality of the clustering solution. Partitioning clustering techniques are based on Euclidean distances, and these distances tend to become less meaningful in high-dimensional spaces. [24].

The proposed work addresses the problem of high dimensional data by proposing a series of different models built on a reduced dimensional space aiming at providing a more appropriate state representation. The results obtained in this study confirm the efficacy of this dimensionality reduction approach, showing that all the proposed models achieved scores higher than those obtained on the original model. Also, our results indicate that the three proposed state representations provide more meaningful state representations than those of the *FULL* model.

The performance of the *CARDIO* model is also consistent with the extremely important role played by the hemodynamic variables in clinical guidelines and severity assessment scores, as well as with their connection with the analyzed treatments that directly affect the cardiovascular system. The importance of hemodynamic variables is further validated by the PCA analysis which ranked them as the major contributors to the first principal component (Fig. 5). The positive impact that the variables have on the model is validated by the performance of the *PCA 80%* and *PCA 15%*, both of these models perform better than the *FULL* model and a high amount of information related to the cited hemodynamic variables is contained on the first principal component included in both models.

Of note, building the Markov Decision Process on a reduced set of 6 variables instead of the full set of 48 variables is extremely helpful to reduce significantly the computational cost, since we have to perform clustering at every model trial.

In previous works we have shown that reducing feature dimensionality increases the value of AI policies [35], [36]. To confirm that the improved performance of the *CARDIO* model was due to the information content of the variables used and not just to dimensionality reduction, we have also developed a *BASELINE* model with the same feature dimensionality as the *CARDIO* model but considering the 6 features with the lowest loading on the first principal component. Table I shows the comparison between the *BASELINE* model and the *CARDIO* model. Despite the slight improvement of the *BASELINE* model, which is clearly due to dimensionality reduction, we can observe a much better performance of the *CARDIO* model, indicating the primary importance of the *CARDIO* variables and confirming that the higher performance is not just due to dimensionality reduction.

The information carried by cardiovascular features could be further enhanced through the use of advanced modeling techniques such as point process modeling [37]. Such methods allow for the robust extraction of instantaneous features from physiological waveforms collected by bedside monitoring equipment. For example, heart-rate variability (HRV) can be easily assessed in continuous time from R wave annotations, and a large number of features related to autonomic nervous system (ANS) dynamics can be consequently extracted at high time resolution. Given that previous studies have established an important role of the ANS in characterizing septic patient outcomes and responses to hemodynamic treatments [38], the addition of ANS features is expected to further improve model performance [6], [39]. Some examples of features that can be extracted are shown in [40].

The implications of our results are quite important from both a technical and a clinical standpoint. First, if cardiovascular variables were sufficient to build and deploy a powerful RL agent, we would only need data from bedside monitoring, thus significantly reducing both human interventions for data collection and data processing resources. Second, the possibility of building an RL agent using only cardiovascular variables could also pave the way for a real-time clinical recommendation system, since the temporal resolution of cardiovascular signals is in the order of milliseconds, while the information coming from other variables, like, for example, those observation points coming from laboratory tests, is more sparse in time and might not reflect the exact situation of the patient at a given time step.

It is important to acknowledge that RL-based approaches come with some limitations. In offline approaches, off-policy evaluation encounters considerable challenges and substantial statistical limitations. The values obtained through these methods do not fully ensure the superiority of the recommended policy over the standard clinical practice [41].

In the current study, our primary objective was to demonstrate the critical information content of variables related to the cardiorespiratory system and to assess the superiority of the *CARDIO* policy compared to other analyzed policies. It is crucial to emphasize that we do not assert that our AI policies will unequivocally outperform clinicians in real-world scenarios. However, we posit that they can serve as valuable tools for clinicians to potentially enhance their practices. Our policies are not intended to replace clinicians but rather to undergo scrutiny and validation by them, thereby enabling the exploration of novel treatment strategies and medical knowledge through the proper management of high-dimensional feature spaces.

Future developments will provide external validation of our policies on a statistically independent dataset and major emphasis will be on explainability and causal inference of our approach, aiming to provide clinicians with a wealth of comprehensive information and tools for analyzing policies.

To partially validate our AI policies, we conducted an analysis of the selected actions and verified their compatibility with recent clinical literature in the management of septic patients. A detailed explanation of our analysis is provided in the following section.

## V. Clinical Implications

**Fig. 6.**
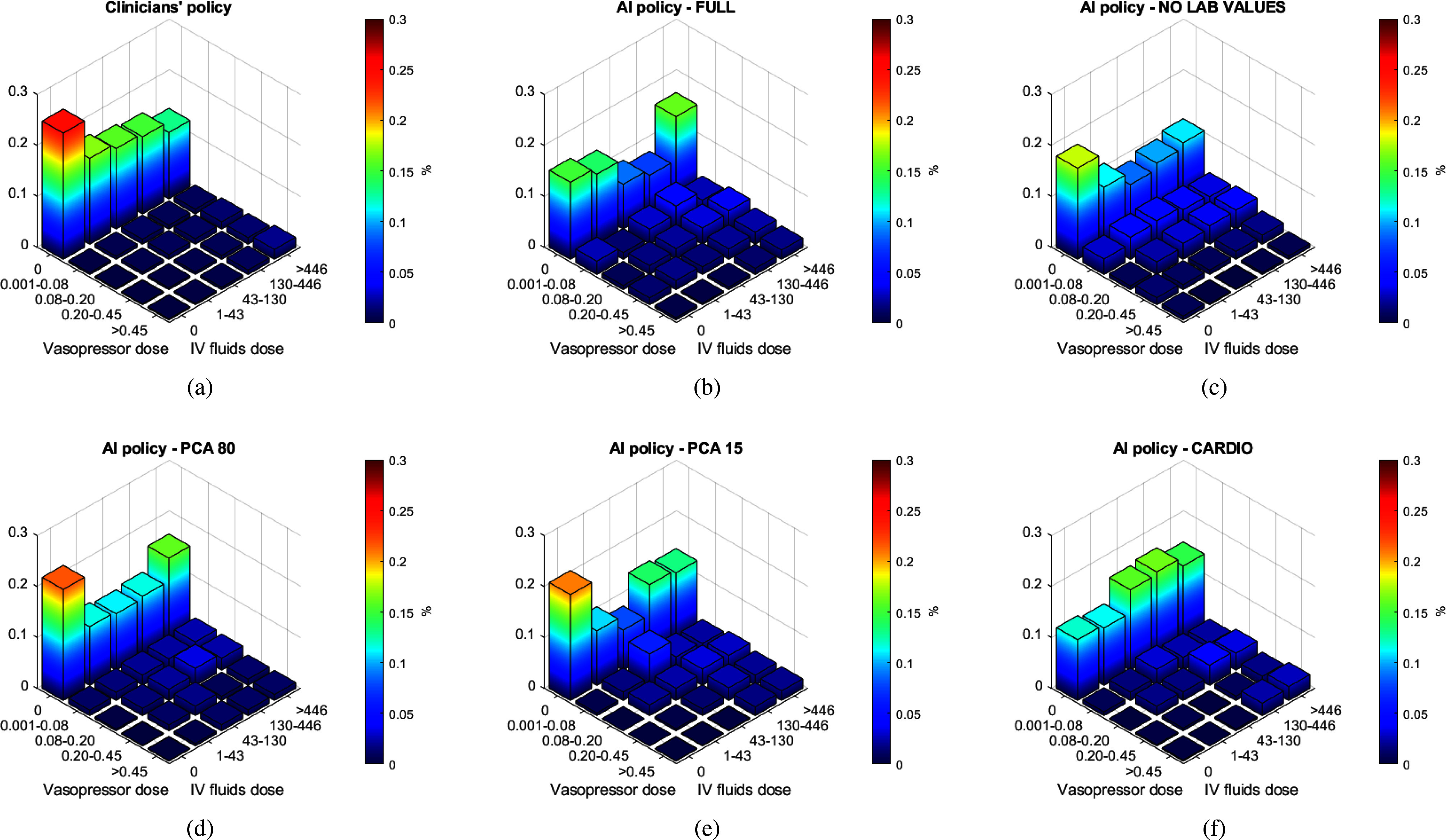
Visualization of action frequencies of clinician and AI policies. Combinations of vasopressors and IV fluids are visualized for a total of 25 bins. Vasopressor dosages are shown as $\mu \text{g}/\text{kg}/\text{min}$ and IV fluid dosages are shown as $\text{mL}/\text{4 h}$. (a) Frequency of actions for the observed clinicians' policy. (b) Frequency of actions for the FULL policy. (c) Frequency of actions for the NO LAB VALUES policy. (d) Frequency of actions for the PCA 80 policy. (e) Frequency of actions for the PCA 15 policy. (f) Frequency of actions for the CARDIO policy.

It is important to provide a rational clinical interpretation of the obtained results. To this extent, Fig. 6 visualizes the action frequencies for the 6 most relevant models. We further provide the quantitative values of all resulting policies in the supplementary material.

In particular, in panel (a) we can observe that clinicians do not give any treatment about 25% of the time, while all AI policies make this choice less frequently. We can also observe that clinicians use vasopressors very sparingly, avoiding them about 85% of the time. These percentages are probably due to the fact that it is now considered standard of care to give several liters of fluid to patients with severe sepsis and septic shock. In fact, most clinicians give an average of about 2 to 3 liters of fluid before administering vasopressors, whereas the optimal timing of vasopressor administration is generally unclear, although some practitioners advocate giving more intravenous fluid in an attempt to avoid the use of vasopressors [22].

Contrary to standard practice, Maitland et al. [42] questioned the safety of the practice of automatically administering liters of fluid. Criticism of the practice also comes from [43], the investigators state that excessive fluid administration in patients with a more permeable endothelium may worsen organ dysfunction and facilitate the development of acute respiratory failure from ARDS. Several authors [44], [45] have also found evidence against current practice, demonstrating that a positive fluid balance was one of the strongest prognostic factors for death in patients with severe sepsis and septic shock.

Evidence against current practice also comes from Waechter et al. [46], the authors find that the lowest mortality rate is achieved when vasopressors are administered between 1 and 6 hours after the onset of sepsis.

The improved outcome, coupled with the increase in vasopressor use, is consistent with the findings of the investigators cited earlier in this section. Therefore, we are led to hypothesize that a possible reason why AI strategies perform better than clinician strategies is that they tend to be more aggressive with vasopressors compared to clinician strategies.

Furthermore, the best performing model, the *CARDIO* model, treats patients more often than both clinicians and all other AI policies. In particular, the frequency of doing nothing is 25% for clinicians, 15% for the *FULL* model, and 12% for the *CARDIO* model.

Another clinical peculiarity associated to the *CARDIO* policy is the increased use of high dosages of vasopressors ($\geq$ 0.45 $\mu \text{g}/\text{kg}/\text{min})$: clinicians and *FULL* model use high dosages 4% of the time while the *CARDIO* model recommends administration of high vasopressor dosages 6% of the times.

## VI. Conclusion

In this work, we have shown that it is possible to build meaningful and effective reinforcement learning models by selecting only cardiovascular variables, and that these models provide a solid foundation for building high-performance decision support systems. When the feature space is reduced from the full dataset to define a model with only cardiorespiratory variables, the performance improves significantly, demonstrating that cardiorespiratory variables alone would be sufficient for the deployment of a reliable RL agent. The obtained results also prove the fact that the improvement obtained with the proposed model is not only due to the reduced dimensionality, but also comes from the intrinsic importance of the cardiorespiratory features. The significance of the proposed model is further confirmed by direct comparison with the *BASELINE* model, which is trained with only the six least informative variables associated with the six lowest loadings on the first principal component. Here, the results show that the proposed model always performs better by all metrics and that this difference in performance can be attributed to the information provided by the cardiovascular variables. Last but not least, findings have allowed us to give a clinical interpretation of the policies. In our study, a successful policy is generally characterized by a higher frequency of vasopressor treatment recommendations compared to clinicians. The *CARDIO* policy suggests increased vasopressor doses beyond those recommended by both the clinician policy and any other AI policy. Since the *CARDIO* policy is the best performing policy, it is interesting to further investigate the effectiveness of the recommended increased vasopressor dosages.

In conclusion, our presented model shows great promise to effectively optimize the treatment strategy for septic patients by relying solely on information obtained from the cardiorespiratory system, however, additional research is needed to further validate our findings and perform a more comprehensive evaluation of the proposed model.

This achievement is a prerequisite for a clinical decision support system that can provide treatment recommendations with a temporal resolution of minutes, as opposed to current applications that provide recommendations on an hourly or longer basis.

*Authors contributions:* C.D. and M.M. contributed to the study design, data extraction and processing, model development, result analysis and interpretation, production of figures and tables, and writing of the manuscript. L.H.L. and R.B. contributed to the interpretation of results and extensively refined the manuscript. C.D. wrote the initial draft of the manuscript. All authors contributed to the critical revision and finalization of the manuscript.

## Supplementary Materials

The supplementary materials provide numerical values for the frequencies of clinician and AI policies. They also display the evolution of the policy value 95% confidence lower bound during training for each model and list the variables included in each model.

Supplementary materials
