# Supplementary material for: A Reinforcement Learning Model for Optimal Treatment Strategies in Intensive Care: Assessment of the Role of Cardiorespiratory Features
Source: IEEE Open J Eng Med Biol. 2024 Feb 19;5:806–15. doi: 10.1109/OJEMB.2024.3367236 (PMC11573419; doi:10.1109/OJEMB.2024.3367236)
Supplement: Supplementary materials [file supp1-3367236.pdf]

Supplementary Materials

A Reinforcement Learning Model for Optimal Treatment Strategies in Intensive Care: Assessment of the Role of Cardiorespiratory Features

Cristian Drudi*, *Student Member, IEEE*, Maximiliano Mollura*, *Member, IEEE*,
Li-wei H. Lehman, *Member, IEEE* and Riccardo Barbieri, *Senior Member, IEEE*

THIS document contains the supplementary materials to: ”A Reinforcement Learning Model for Optimal Treatment Strategies in Intensive Care: of the Role of Cardiorespiratory Features” by Drudi et al.

I. FREQUENCY OF ACTIONS SELECTED BY POLICIES

Clinicians	IV_1	IV_2	IV_3	IV_4	IV_5
VP_1	0.1511	0.1374	0.0879	0.0758	0.1611
VP_2	0.0260	0.0131	0.0294	0.0452	0.0268
VP_3	0.0079	0.0173	0.0212	0.0317	0.0356
VP_4	0.0135	0.0177	0.0148	0.0206	0.0237
VP_5	0.0043	0.0028	0.0049	0.0119	0.0183

TABLE I. Normalized frequencies of action chosen by the clinicians policy

FULL	IV_1	IV_2	IV_3	IV_4	IV_5
VP_1	0.2477	0.1684	0.1578	0.1514	0.1301
VP_2	0.0033	0.0061	0.0096	0.0076	0.0093
VP_3	0.0021	0.0050	0.0093	0.0105	0.0140
VP_4	0.0018	0.0035	0.0047	0.0076	0.0129
VP_5	0.0021	0.0028	0.0044	0.0087	0.0194

TABLE II. Normalized frequencies of actions chosen by the *FULL* model

NO LAB	IV_1	IV_2	IV_3	IV_4	IV_5
VP_1	0.1788	0.1116	0.0872	0.0996	0.1099
VP_2	0.0303	0.0432	0.0457	0.0379	0.0337
VP_3	0.0072	0.0246	0.0312	0.0360	0.0372
VP_4	0.0138	0.0095	0.0085	0.0154	0.0177
VP_5	0.0074	0.0020	0.0011	0.0038	0.0067

TABLE III. Normalized frequencies of actions chosen by the *NO LAB* model

PCA_{80}	IV_1	IV_2	IV_3	IV_4	IV_5
VP_1	0.2179	0.1150	0.1106	0.1153	0.1605
VP_2	0.0106	0.0156	0.0201	0.0224	0.0257
VP_3	0.0031	0.0142	0.0219	0.0332	0.0235
VP_4	0.0009	0.0113	0.0184	0.0081	0.0121
VP_5	0.0008	0.0045	0.0117	0.0112	0.0116

TABLE IV. Normalized frequencies of actions chosen by the PCA_{80} model

In this section we illustrate the normalized frequencies of actions selected by the six most relevant policies illustrated in the paper in Figure 6 using tables.

In all the tables IV_1 corresponds to a median dosage of intravenous fluid of 0 mL/h, IV_2 corresponds to a median dosage of intravenous fluid of 30 mL/h, IV_3 corresponds to a median dosage of intravenous fluid of 77 mL/h, IV_4 corresponds to a median dosage of intravenous fluid of 250 mL/h and IV_5 corresponds to a median dosage of intravenous fluid of 808 mL/h.

PCA_{15}	IV_1	IV_2	IV_3	IV_4	IV_5
VP_1	0.2077	0.1068	0.0786	0.1379	0.1318
VP_2	0.0051	0.0156	0.0201	0.0224	0.0257
VP_3	0.0000	0.0207	0.0238	0.0319	0.0288
VP_4	0.0000	0.0039	0.0130	0.0247	0.0200
VP_5	0.0007	0.0007	0.0014	0.0173	0.0188

TABLE V. Normalized frequencies of actions chosen by the PCA_{15} model

CARDIO	IV_1	IV_2	IV_3	IV_4	IV_5
VP_1	0.1192	0.1126	0.1577	0.1631	0.1450
VP_2	0.0073	0.0163	0.0312	0.0253	0.0230
VP_3	0.0000	0.0183	0.0168	0.0393	0.0331
VP_4	0.0025	0.0050	0.0015	0.0084	0.0177
VP_5	0.0021	0.0000	0.0031	0.0243	0.0272

TABLE VI. Normalized frequencies of actions chosen by the *CARDIO* model

VP_1 corresponds to a median dosage of vasopressors of 0 $\mu g/kg/min$, VP_2 corresponds to a median dosage of vasopressors of 0.04 $\mu g/kg/min$, VP_3 corresponds to a median dosage of vasopressors of 0.135 $\mu g/kg/min$, VP_4 corresponds to a median dosage of vasopressors of 0.27 $\mu g/kg/min$ and VP_5 corresponds to a median dosage of vasopressors of 0.8 $\mu g/kg/min$.

II. EVOLUTION OF THE 95% CONFIDENCE LOWER BOUND

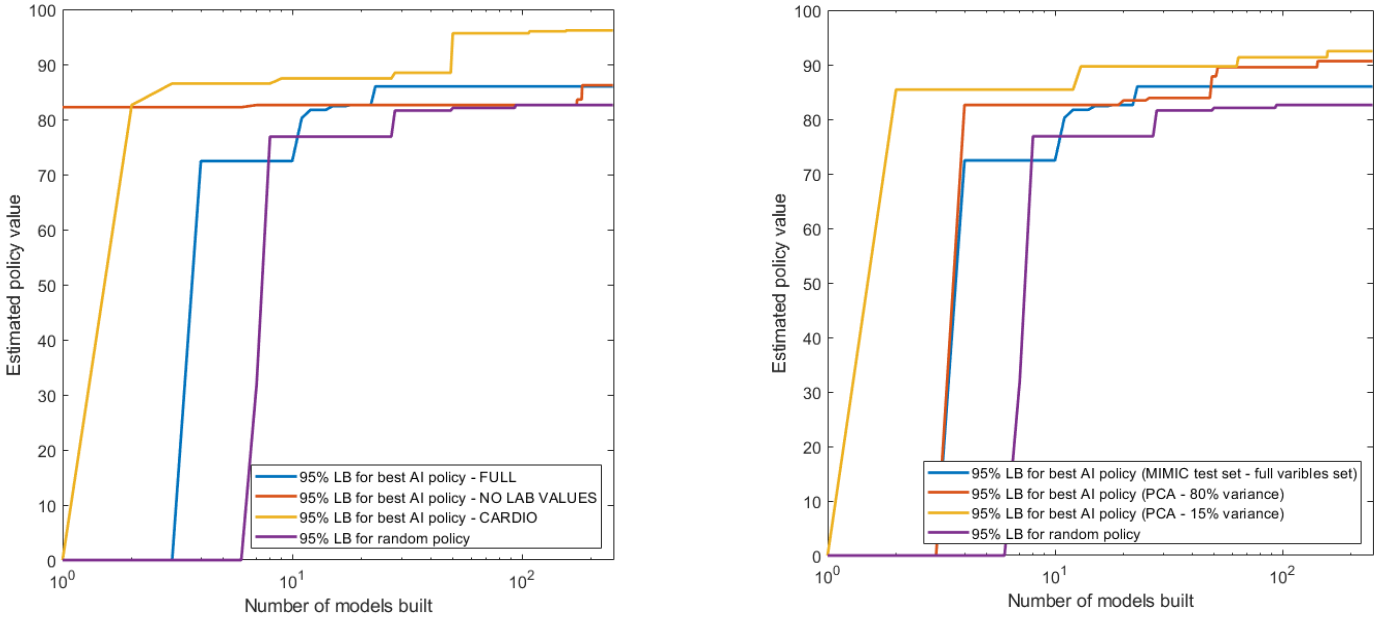

(a) Evolution of the 95% confidence Lower Bound of the *FULL*, *NO LAB*, *CARDIO* and *RAND* models.

(b) Evolution of the 95% confidence Lower Bound of the *FULL*, *PCA 80%*, *PCA 15%* and *RAND* models.

Fig. 1. Evolution of the 95% confidence Lower Bound of the 5 most relevant AI models discussed in the paper. We compare them with the *RAND* model that serves as baseline.

In Figure 1 we illustrate the evolution of the 95% confidence Lower Bound over training of the five most relevant AI models illustrated in the paper in Figure 6.

The estimates were obtained using the Weighted Importance Sampling (WIS) estimator combined with the bootstrapping technique.

III. INCLUDED VARIABLES

Below is a detailed list of the included features for each model:

FEATURE	Full	NOLAB	PCA80%	PCA15%	CARDIO
DEMOGRAPHICS					
Age	Yes	Yes	Yes	Yes	No
Gender	Yes	Yes	Yes	Yes	No
Weight	Yes	Yes	Yes	Yes	No
Readmission to intensive care	Yes	Yes	Yes	Yes	No
Elixhauser score	Yes	Yes	Yes	Yes	No
VITAL SIGNS					
Modified SOFA*	Yes	Yes	Yes	Yes	No
SIRS	Yes	Yes	Yes	Yes	No
Glasgow coma scale	Yes	Yes	Yes	Yes	No
Heart rate	Yes	Yes	Yes	Yes	Yes
Systolic blood pressure	Yes	Yes	Yes	Yes	Yes
Mean blood pressure	Yes	Yes	Yes	Yes	No
Diastolic blood pressure	Yes	Yes	Yes	Yes	Yes
Shock index	Yes	Yes	Yes	Yes	Yes
Respiratory rate	Yes	Yes	Yes	Yes	No
SpO2	Yes	Yes	Yes	Yes	Yes
Temperature	Yes	Yes	Yes	Yes	No
LAB VALUES					
Potassium	Yes	No	Yes	Yes	No
sodium	Yes	No	Yes	Yes	No
chloride	Yes	No	Yes	Yes	No
Glucose	Yes	No	Yes	Yes	No
BUN	Yes	No	Yes	Yes	No
Creatinine	Yes	No	Yes	Yes	No
Magnesium	Yes	No	Yes	Yes	No
Calcium	Yes	No	Yes	Yes	No
Ionized calcium	Yes	No	Yes	Yes	No
Carbon dioxide	Yes	No	Yes	Yes	No
SGOT	Yes	No	Yes	Yes	No
SGPT	Yes	No	Yes	Yes	No
Total bilirubin	Yes	No	Yes	Yes	No
Albumin	Yes	No	Yes	Yes	No
Hemoglobin	Yes	No	Yes	Yes	No
White blood cells count	Yes	No	Yes	Yes	No
Platelets count	Yes	No	Yes	Yes	No
PTT	Yes	No	Yes	Yes	No
PT	Yes	No	Yes	Yes	No
INR	Yes	No	Yes	Yes	No
pH	Yes	No	Yes	Yes	No
PaO2	Yes	No	Yes	Yes	No
PaCO2	Yes	No	Yes	Yes	No
Base excess	Yes	No	Yes	Yes	No
Bicarbonate	Yes	No	Yes	Yes	No
Lactate	Yes	No	Yes	Yes	No
PaO2/FiO2 ratio	Yes	No	Yes	Yes	No
VENTILATION					
Mechanical ventilation	Yes	Yes	Yes	Yes	Yes
FiO2	Yes	Yes	Yes	Yes	No
MEDICATIONS					
Current IV fluid intake over 4h	Yes	Yes	Yes	Yes	No
Max vasopressor dose over 4h	Yes	Yes	Yes	Yes	No
Urine output over 4h	Yes	Yes	Yes	Yes	No
Cumulated fluid balance**	Yes	Yes	Yes	Yes	No

TABLE VII. Description of the variables included in the datasets. INR: International Normalized Ratio; * Modified SOFA: SOFA based on value in the current 4h time step; PEEP: Positive End Expiratory Pressure; PT: Prothrombin Time; PTT: Partial Thromboplastin Time; SIRS: Systemic Inflammatory Response Syndrome; Shock index: systolic blood pressure/heart rate; **: Cumulated fluid balance since admission (includes preadmission data when available)
